# Bibliometric analysis of global research on human organoids

**DOI:** 10.1016/j.heliyon.2024.e27627

**Published:** 2024-03-10

**Authors:** Huanyu Li, Daofeng Wang, Cheong Wong Ho, Dan Shan

**Affiliations:** a Department of Pharmacology, School of Pharmacy, China Medical University, Shenyang, Liaoning, 110122, China; b Liaoning Key Laboratory of Molecular Targeted Anti-Tumor Drug Development and Evaluation, Liaoning Cancer Immune Peptide Drug Engineering Technology Research Center, Key Laboratory of Precision Diagnosis and Treatment of Gastrointestinal Tumors (China Medical University), Ministry of Education, Shenyang, Liaoning, 110122, China; c Sports Medicine Service, Capital Medical University Affiliated Beijing Jishuitan Hospital, No. 31, Xinjiekou East Street, Beijing, 10035, China; d Clinical Science Institute, University Hospital Galway, University of Galway, Ireland; e Regenerative Medicine Institute, School of Medicine, National University of Ireland Galway, Ireland

**Keywords:** Human organoid, Bibliometric analysis, Biobanking, Precision medicine, Disease modeling

## Abstract

The emergence and rapid development of human organoids have provided the possibility to replace animal models in treating human diseases. Intelligence studies help focus on research hotspots and address key mechanistic issues. Currently, few comprehensive studies describe the characteristics of human organoid research. In this study, we extracted 8,591 original articles on organoids from the Web of Science core collection database over the past two decades and conducted intelligence analysis using CiteSpace. The number of publications in this field has experienced rapid growth in the last ten years (almost 70-fold increase since 2009). The United States, China, Germany, Netherlands, and UK have strong collaborations in publishing articles. Clevers Hans, Van Der Laan, Jason R Spence, and Sato Toshiro have made significant contributions to advancing progress in this field. Clustering and burst analysis categorized research hotspots into tissue model and functional construction, intercellular signaling, immune mechanisms, and tumor metastasis. Organoid research in highly cited articles covers four major areas: basic research (38%), involving stem cell developmental processes and cell-cell interactions; biobanking (10%), with a focus on organoid cultivation; precision medicine (16%), emphasizing cell therapy and drug development; and disease modeling (36%), including pathogen analysis and screening for disease-related genetic variations. The main obstacles currently faced in organoid research include cost and technology, vascularization of cells, immune system establishment, international standard protocols, and limited availability of high-quality clinical trial data. Future research will focus on cost-saving measures, technology sharing, development of international standards, and conducting high-level clinical trials.

## 1 Introduction

The application of classical cell lines and animal model systems has led to many successes in contemporary biomedical developments, such as deepening human understanding of cell signaling pathways [1,2], drug targets [3,4], and cancer [5] and infectious disease mechanisms [6]. However, these systems have been studied along a single pipeline and have never been able to simulate biological processes specific to the human body [[7], [8], [9]]. The truth is that some important aspects of brain development, metabolism, and drug efficacy testing of organisms mimicked in animals cannot yet be fully and reliably extended to humans [10]. Real-life examples are the many drugs that perform well in cancer models that end up failing in clinical trials [[7], [8], [9]]. The transfer of findings from model systems to humans has become a big hurdle in the drug discovery process. Given this, the rise of human in vitro 3D cell culture techniques using stem cells from various organs has grabbed significant attention due to its potential in tackling these limitations [11,12].

Organoid is a spatially structured tissue analog formed by in vitro three-dimensional (3D) culture of adult (ASC) or pluripotent stem cells (PSC). They are also called “Mini-Organs” because they have highly similar histological characteristics to their human counterparts and can reproduce the physiological functions of the organ [12,13]. Human organoids have been employed for investigating infectious diseases, genetic disorders, and cancers by manipulating human stem cells genetically and generating organoids directly from biopsy samples taken from patients [13,14]. While animals are typically subjected to harmful conditions or have disease-causing genes manipulated to create animal models, organoid models can be created straight from affected patients without needing prior knowledge of the specific genes responsible for the disease [3,9]. Human organoid cultures bring forth a whole host of potential advantages compared to animal models: organoids deliver speedier and more dependable outcomes, are more easily accessible, present a truer reflection of human tissue, and offer a greater quantity of material than their animal counterparts [10]. In January this year, the FDA issued new regulations recognizing new drugs for approval without animal testing (https://www.science.org/content/article/fda-no-longer-needs-require-animal-tests-human-drug-trials). This is a groundbreaking move in the field of drug development. Human organoid research has gained wide attention and rapid development in recent years. It is of great research and translation significance to carry out intelligence research and hotspot exploration for human organoid research.

The bibliometric analysis focuses on the publication system and its characteristics as the research objective, utilizing mathematical and statistical methods to examine the distribution structure, quantitative relationships, and research topics within literature data. This analysis assists researchers in assessing publication trends and identifying hot topics within a specific field [15,16]. So far, researchers have used bibliometric methods to analyze the development hotspots and future trends of retinal organoids [17] and organ-on-a-chip research [18]. These studies provide unique perspectives on the evolution of organoids in different tissues and organs from the aspects of research hotspots, influencing scientific fields, and global trends. They guide the establishment of disease models and biobanks, providing possible solutions for cancer research and various genetic disorders. Our objective is to identify publications that delve into the exploration and application of organoids, and provide a detailed characterization of these articles.

## 2 Methods

### 2.1 Articles search and selection

On January 10, 2024, we conducted an extensive search using the Web of Science Core Collection database to gather a collection of published studies pertaining to organoids. To retrieve publications, we utilized specific search terms and topics: (TI=(organoid*) OR AB=(organoid*)). We further filtered the results by selecting “Article” as the file type, specifying “English” as the language, and setting the publication period from January 1, 2003, to January 10, 2024. This comprehensive search strategy yielded a total of 8,692 publications. Through this rigorous process, we assembled a compilation of studies exploring and applying organoids over nearly two decades. For a detailed record of our search history, please refer to the supplementary table. Two reviewers, Huanyu Li and Daofeng Wang, conducted separate searches, identification, and analysis of the articles. In the event of encountering ambiguous research, the final decision will be entrusted to a third researcher (Ho Cheong Wong). After careful consideration, we excluded 101 conferences and proceeded with the analysis of 8,591 original articles.

### 2.2 Bibliometric analysis

The Web of Science's inherent capabilities were harnessed to delve into the essential attributes of the publications. Each article underwent meticulous scrutiny, extracting crucial details such as publication year, authors, institutions, countries, H-index, publication journal, journal citation reports, and the keywords encapsulated within the article. The H-index, a widely acknowledged metric for assessing academic productivity, has been thoroughly examined across various medical and surgical specialties. These studies have consistently showcased strong correlations between elevated H-indices and academic progression, as well as the attainment of prestigious accolades from the National Institutes of Health [19]. Furthermore, this study delved into the features of the top 100 most cited articles associated with the organoid. Articles that garner substantial citations are considered integral to their respective domains. Hence, extensively referenced publications can offer critical insights into research patterns and significant breakthroughs within a particular discipline [20].

### 2.3 Data visualization and knowledge-map analysis

We employed Microsoft Office Excel and GraphPad Prism (version 8.3.0) to store data and present annual publication trends, institutional contributions, and funding distributions, respectively. For visualizing co-authorship, co-citation, and co-occurrence analyses, we utilized CiteSpace (version 6.2.R4), a widely used visual analysis software in bibliometric studies. Developed by Chaomei Chen, CiteSpace offers a multidimensional perspective to explore and visualize research hotspots and trends within a specific field by dissecting clusters of literature [16].

Collaboration network analysis enables the evaluation of collaborations between countries, institutions, and authors. Co-cited references refer to two or more references (journals, authors) that are cited by one or more articles simultaneously, indicating their relevance and influence in the field [15,21]. Through clustering analysis of collaboration network and co-citation results, we can reveal the research categories and directions within each study cluster. Furthermore, we employed co-occurrence analysis (commonly known as keyword analysis) to group keywords from various studies, enabling us to identify and anticipate research hotspots in the field [21].

### 2.4 Statistical analysis of the top 100 cited publications

We utilized SPSS (version 26.0, IBM, Armonk, NY, USA) to depict the data distribution and examine the correlation between JIF (journal impact factor) and CIF (number of citations/impact factor) ratio. The normality of continuous data was assessed using the Kolmogorov-Smirnov test. Continuous variables that passed the normality test were presented as means and standard deviations (SD), while those that did not conform to normality were presented as medians.

## 3 Results

A total of 8,591 original articles focusing on organoid research were selected for further analysis. The annual publication count for organoid-related studies from 2003 to 2023 is displayed in Fig. 1. The curve depicting the number of publications can be divided into three distinct periods: a period of relative stability (P1, 2003–2008), a phase of gradual growth (P2, 2009–2016), and an accelerated growth period (P3, 2017–2023). After 2009, the number of published studies exploring organoids experienced a considerable surge, with over 1770 articles per year in 2023, representing an almost 70-fold increase compared to 2009. The trend suggests that the number of publications will continue to rise. The supplementary figure showcases the top 15 study categories and funding sources based on the overall number of publications.

**Fig. 1:**
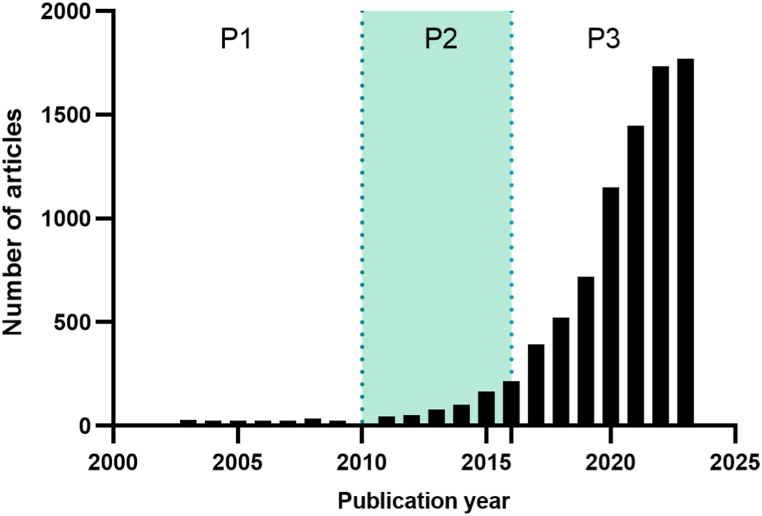
Annual publication count on organoids from 2003 to 2023.

### 3.1 Countries, institutions, and authors

**Fig. 2:**
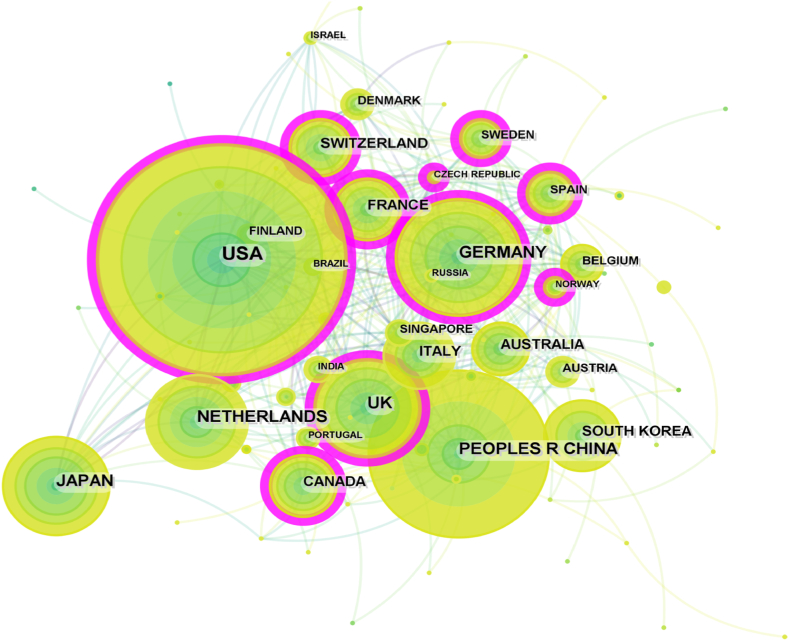
Collaborative network analysis of countries involved in organoid research.

The publications encompassed a wide range of contributors, including 96 countries or regions, 6,042 institutions, and 52,758 authors. The United States had the highest number of contributions (*n* = 3,712, 43%), followed at a distance by China (*n* = 1,693, 20%), Germany (*n* = 1,011, 12%), Japan (*n* = 827, 10%), and the Netherlands (*n* = 812, 9%) (Table 1). These countries accounted for 94% of the total contributions. When considering the total number of citations and the average citation per publication (citation/publication ratio), the United States stood out with 165,981 citations (average of 44.7 citations) and an impressive H-index of 182, surpassing all other countries by a significant margin. Although the Netherlands and England had relatively fewer publications, their citation/publication ratios (77 and 56.8, respectively) were relatively high among the top ten countries. While China had numerous publications, its citation/publication ratio was relatively lower (18.8). The collaboration network analysis depicted in Fig. 2 revealed that the United States, China, Germany, the Netherlands, England, and Japan exhibited substantial output volumes and closely interconnected collaborative networks with each other.

**Table 1:** The top 10 productive countries/regions and institutions related to organoid research.

Country	No. (%)	Total of citations	Average citations	H-index	Institution	No	Total of citations	Average citations	H-index
USA	3,712 (43)	165,981	44.71	182	University of California System	477	25,898	54.29	82
China	1,693 (20)	31,900	18.84	78	Harvard University	467	30,591	65.51	88
Germany	1,011 (12)	33,047	32.69	93	Utrecht University	360	47,846	132.91	94
Japan	827 (10)	27,932	33.78	76	Helmholtz Association	280	8,257	29.49	49
Netherlands	812 (9)	62,527	77	108	Chinese Academy of Sciences	271	6,366	23.49	43
England	688 (8)	39,105	56.84	87	Royal Netherlands Academy of Arts Sciences	246	47,609	193.53	93
Italy	429 (5)	15,112	35.23	59	Hubrecht Institute Knaw	239	47,211	197.54	92
South Korea	419 (5)	9,923	23.68	47	University of Texas System	228	8,582	37.64	49
Canada	385 (4)	17,199	44.67	60	Institut National De La Sante Et De La Recherche Medicale Inserm	221	7,005	31.7	41
France	352 (4)	10,205	28.99	50	University of London	214	10,284	48.06	52

**Fig. 3:**
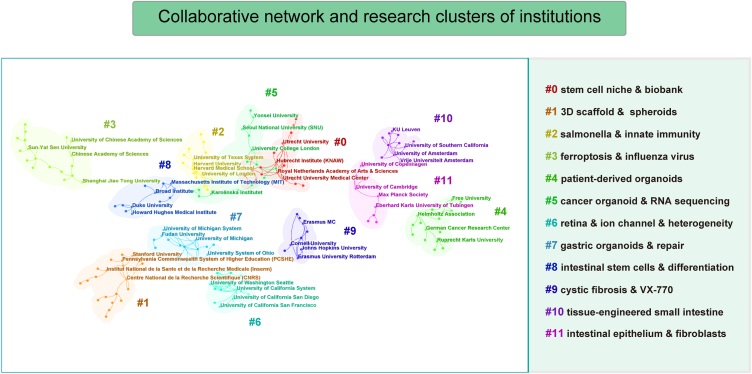
Collaborative network analysis of institutions engaged in organoid research and their respective clusters based on research focus.

The top 10 institutions of the overall number of articles were summarized in Table 1. The University of California System published most studies (*n* = 477) with a citation/publication ratio of 54.3 and an H-index of 82, followed by Harvard University (467 articles, average citations: 65.5, H-index: 88), and Utrecht University (360 articles, average citations: 132.9, H-index: 94). The institutions to note are that Royal Netherlands Academy of Arts Sciences and Hubrecht Institution Knaw have a moderate number of publications, but they are of good quality (average citations: 193.5 and 197.5; H-index: 93 and 92, respectively). In this study, we focused on investigating the collaborative networks and clustering of research directions among institutions. Fig. 3 illustrates the division of institutions involved in studies on organoid exploration into 11 distinct clusters, each with its specific research orientation. For instance, Harvard University and its affiliated institutions primarily concentrated on conducting studies related to innate immunity and salmonella. Similarly, the Royal Netherlands Academy of Arts and Sciences and its collaborative institutions primarily focused on research related to stem cell niche and biobanking.

**Fig. 4:**
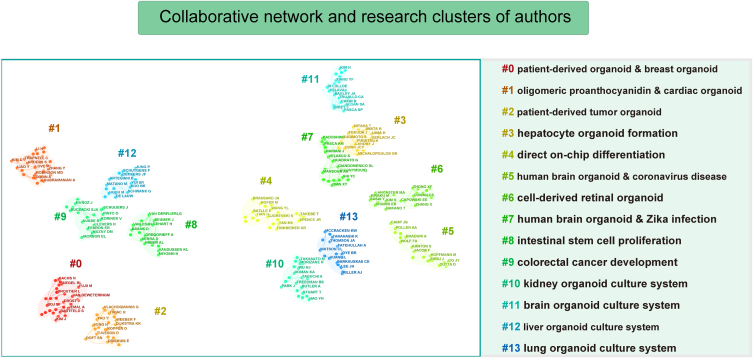
Collaborative network analysis of co-cited authors in organoid research and their corresponding clusters based on research direction.

Table 2 displays the top 10 authors based on the total number of publications and the top 10 co-cited authors. Hans Clevers from the Royal Netherlands Academy of Arts and Sciences emerged as the leading researcher with the highest number of published studies (*n* = 208). His articles had a remarkable citation/publication ratio of 219.7 and an impressive H-index of 91. Toshiro Sato, despite publishing a smaller number of articles, exhibited the highest citation/publication ratio (398.3), indicating the exceptional quality of his work. Fig. 4 visualizes the collaborative networks and clustering of research directions among authors. It reveals that all authors can be categorized into 14 distinct clusters, each focusing on the development and testing of various types of organoids within their respective research networks.

**Table 2:** Top 10 productive authors and co-cited authors related to organoid research.

Authors	Institution	No.	Citation (per-citation)	H-index	Co-cited authors	Institution	Citations
Clevers, Hans	Royal Dutch Academy of Science (KNAW) and University Medical Center Utrecht	208	4,5701 (219.72)	91	Sato, Toshiro	Department of Organoid Medicine, Keio University School of Medicine, Tokyo, Japan.	2033
Spence, Jason R	Gastroenterology and Hepatology, Michigan Medicine at the University of Michigan	66	6209 (94.08)	33	Lancaster, Madeline A	Medical Research Council Laboratory of Molecular Biology	1242
Van Der Laan, Luc	Department of Surgery, Erasmus MC-University Medical Center	53	3714 (70.08)	24	Barker, Nick	A*STAR Institute of Medical Biology, Singapore.	843
Sato, Toshiro	Department of Organoid Medicine, Keio University School of Medicine	43	17,127 (398.3)	32	Clevers, Hans	Royal Dutch Academy of Science (KNAW) and University Medical Center Utrecht	767
Verstegen, Monique, MA	Erasmus University Rotterdam.	41	3230 (78.78)	19	Huch, Meritxell	Center for Systems Biology Dresden and Cluster of Excellence Physics of Life	622
Wells, James M.	Cincinnati Children S Hospital Medical Center	37	5075 (137.16)	21	Drost, Jarno	Princess Máxima Center for Pediatric Oncology, Utrecht	567
Roberts, Lewis	Utrecht University	33	8974 (271.94	26	Van de Wetering, Willine J	Royal Netherlands Academy of Arts and Sciences (KNAW) and UMC Utrecht.	526
Peppelenbosch, Maikel P.	Department of Gastroenterology and Hepatology, Erasmus MC-University Medical Center	32	904 (28.25)	17	Norman Sachs	Royal Netherlands Academy of Arts and Sciences and University Medical Center Utrecht	504
Cuppen, Edwin	Center for Molecular Medicine and Oncode Institute, University Medical Centre Utrecht	32	10,300 (321.88)	26	Qian, Xuyu	Johns Hopkins University School of Medicine	500
Pan, Qiuwei	Erasmus University Rotterdam	31	809 (26.1)	20	Sylvia F Boj	Hubrecht Organoid Technology (HUB)	461

### 3.2 Journals and references

All of the organoids studies were published in 1,217 journals. The majority of the articles were contributed by Scientific Reports (266 articles, 24.9 average citations), followed by Nature Communications (265 articles, 40.5 average citations), Jove-Journal of Visualized Experiments (163 articles, 8.2 average citations), and Cell Reports (158 articles, 45.6 average citations) (Table 3). The Proceedings of the National Academy of Sciences of the United States of America published a relatively low number of publications but yielded the highest citation/publication ratio of 64.5.

**Table 3:** The top 10 journals and co-cited journals related to organoid research.

Journal	Number	Citation (per citation)	IF (2022)	JCR (2022)	Co-cited journal	Citation	IF (2022)	JCR (2022)
Scientific Reports	266	6,615 (24.87)	4.6	Q2	Nature	6,350	64.8	Q1
Nature Communications	265	10,737 (40.52)	16.6	Q1	Cell	5,514	64.5	Q1
JoVE-Journal of Visualized Experiments	163	1,329 (8.15)	1.2	Q3	Proceedings of the National Academy of Sciences of the United States of America	5,476	11.1	Q1
Cell Reports	158	7,203 (45.59)	8.8	Q1	Science	4,751	56.9	Q1
International Journal of Molecular Sciences	155	1,222 (7.88)	5.6	Q2	Nature Communications	4,103	16.6	Q1
Stem Cell Reports	149	56,75 (38.09)	5.9	Q1	PLoS One	4,027	3.7	Q2
Cells	121	1,045 (8.64)	6	Q2	Cell Stem Cell	3,708	23.9	Q1
PLoS One	120	2,996 (24.97)	3.7	Q2	Scientific Reports	3,527	4.6	Q2
Proceedings of the National Academy of Sciences of the United States of America	117	7,547 (64.5)	11.1	Q1	Nature Medicine	3,065	82.9	Q1
Cancers	113	1,008 (8.92)	5.2	Q2	Nature Protocol	2,899	14.8	Q1

**Fig. 5:**
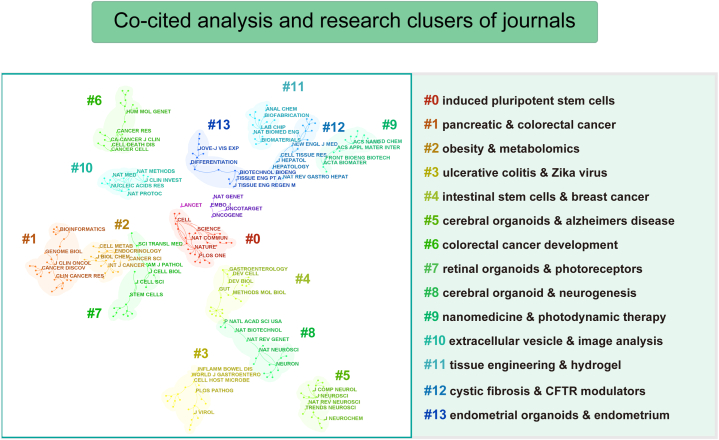
Visualization of co-cited analysis of journals in organoid research and their corresponding clusters based on research direction.

**Fig. 6:**
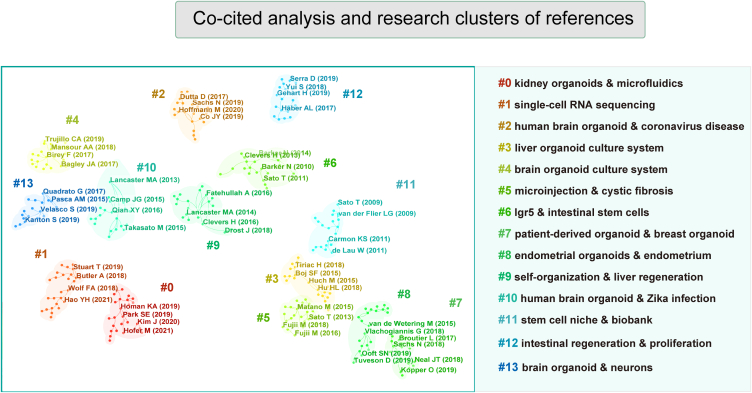
Visualization of co-cited analysis of references in organoid research and their corresponding clusters based on research direction.

**Fig. 7:**
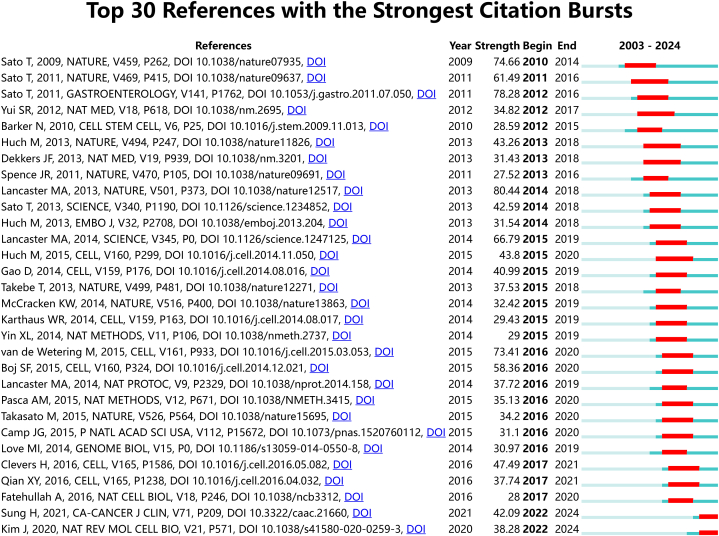
Burst analysis of co-cited references.

The most highly cited articles were published in co-cited journals, with Nature leading the way with 6,350 citations, followed by Cell with 5,514 citations, Proceedings of the National Academy of Sciences of the United States of America (PNAS) with 5,476 citations, and Science with 4,751 citations. Table 3 provides a summary of the impact factors and Journal Citation Reports (JCR) categories for both journals and co-cited journals. Fig. 5 presents a visual representation of the co-citation analysis of journals, showcasing clusters based on research directions. These cited journals were categorized into 14 distinct groups, each emphasizing specific research preferences within the field. The top 5 most frequently co-cited references involved Clevers H (2016) [22], Vlachogiannis G (2018) [23], Qian XY (2016) [24], Sachs N (2018) [25], and van de Wetering M (2015) [26]. Fig. 6 provides a summary of the clustering analysis based on research directions and detailed characterizations of co-cited references across 14 categories. To identify the research progress, journal burst analysis in CiteSpace was utilized, highlighting emerging references within specific periods. Fig. 7 showcases the top 30 references with the most significant citation bursts, as determined by burst intensity.

### 3.3 Keywords

**Fig. 8:**
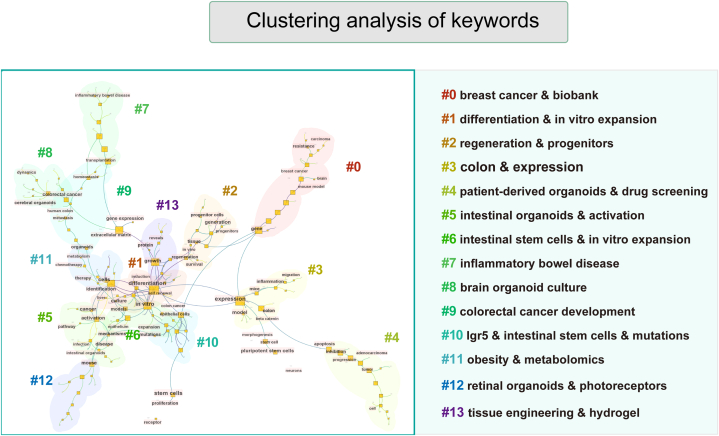
Burst analysis of keywords.

**Fig. 9:**
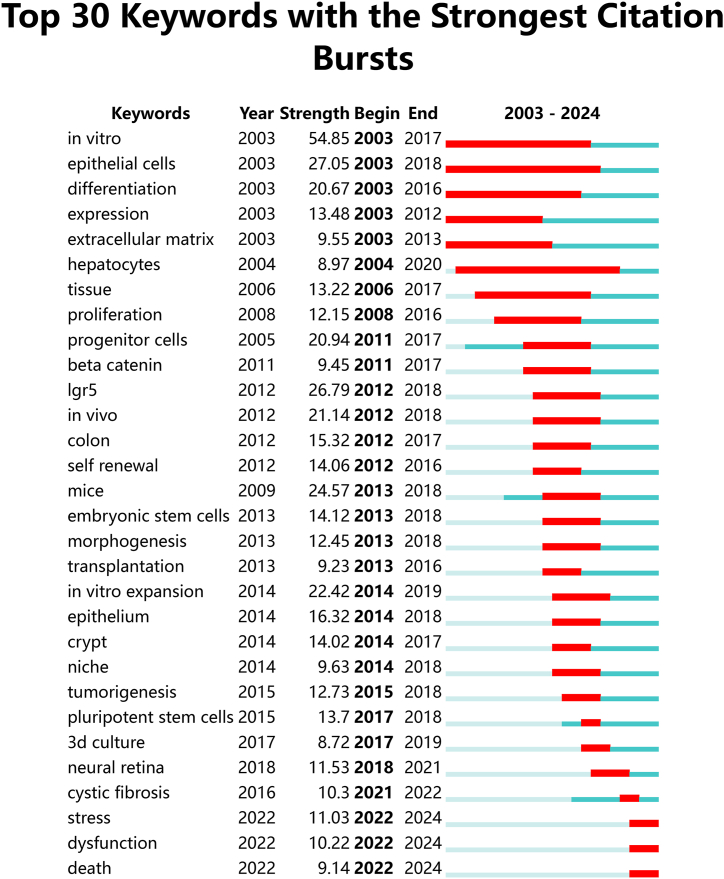
Visualization mapping and clusters of keywords in organoid research and their corresponding clusters based on research direction.

29,026 keywords were extracted and further classified into 14 clusters (Fig. 8) with the help of co-occurrence analysis in CiteSpace: #1 breast cancer & biobank, #2 differentiation & in vitro expansion, #3 regeneration & progenitors, #4 colon & expression, #5 patient-derived organoids & drug screening, #6 intestinal organoids & activation, #7 intestinal stem cells & in vitro expansion, #8 inflammatory bowel disease, #9 brain organoid culture, #10 colorectal cancer development, #11 Igr5 & intestinal stem cells & mutations, #12 obesity & metabolomics, #13 retinal organoids & photoreceptors, and #14 tissue engineering & hydrogel. Fig. 9 showcases the top 30 references with the most significant citation bursts, as determined by burst intensity.

### 3.4 Systematic review of highly cited articles

**Fig. 10:**
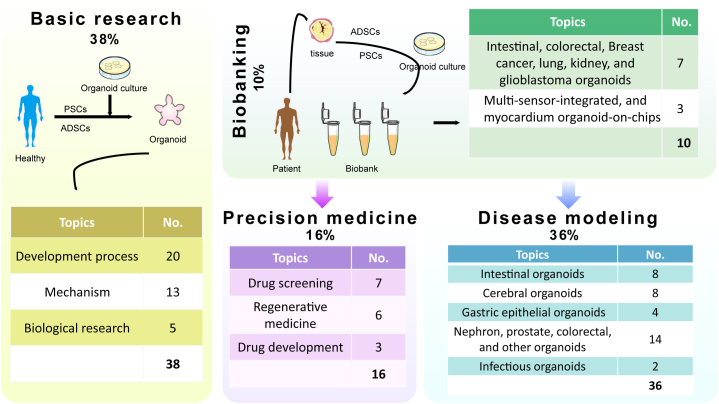
Key topics addressed in the top 100 highly cited articles.

Publication with a high number of citations has far-reaching implications for the advancement of a specific domain [27]. Here, we also featured highly cited published articles related to organoids. The highly cited articles received a range of citations, from 4,411 to 378 (median: 552). Table 4 displays the top 20 most cited publications on organoids, along with their corresponding citation frequencies. The table includes information such as the article title, study type, citations over the years, first author, and journal details. The article with the highest total citation was published in Nature in 2009 by Sato Toshiro et al. [28]. Four main topics were summarized from the top 100 highly cited articles: basic research (38%), disease modeling (36%), precision medicine (16%), and biobanking (10%). Basic research is based on culturing organ-like structures using healthy human tissues for studying cellular-level mechanisms. The biological banking, derived from pathological tissues, is utilized for cell therapy, drug development, genetic engineering, and various precision medicine fields, as well as modeling and therapeutic analysis of different disease models (Fig. 10).

**Table 4:** The 20 most-cited organoid articles ranked in order of the number of citations received.

Rank	Article	Topics	No. of citations	Citations over year			Author (first)	Journal
				P1	P2	P3		
1	Single Lgr5 stem cells build crypt-villus structures in vitro without a mesenchymal niche	Disease modeling	4,411	17	870	3,518	Sato, Toshiro	Nature
2	Cerebral organoids model human brain development and microcephaly	Disease modeling	2,920	0	257	2,654	Lancaster, Madeline A.	Nature
3	Long-term Expansion of Epithelial Organoids From Human Colon, Adenoma, Adenocarcinoma, and Barrett's Epithelium	Disease modeling	2,308	0	240	2,058	Sato, Toshiro	Gastroenterology
4	Paneth cells constitute the niche for Lgr5 stem cells in intestinal crypts	Disease modeling	1,761	0	566	1,194	Sato, Toshiro	Nature
5	Inhibition of SARS-CoV-2 Infections in Engineered Human Tissues Using Clinical-Grade Soluble Human ACE2	Basic research	1,461	0	0	1,461	Monteil, Vanessa	Cell
6	Prospective Derivation of a Living Organoid Biobank of Colorectal Cancer Patients	Biobanking	1,432	0	24	1,406	Van de Wetering, Marc	Cell
7	Organoid Models of Human and Mouse Ductal Pancreatic Cancer	Disease modeling	1,307	0	36	1,268	Boj, Sylvia F.	Cell
8	Distinct populations of inflammatory fibroblasts and myofibroblasts in pancreatic cancer	Basic research	1,299	0	0	1,294	Ohlund, Daniel	Journal of Experimental Medicine
9	Directed differentiation of human pluripotent stem cells into intestinal tissue in vitro	Disease modeling	1,299	0	273	1,022	Spence, Jason R.	Nature
10	Brain-Region-Specific Organoids Using Mini-bioreactors for Modeling ZIKV Exposure	Disease modeling	1,296	0	0	1,294	Qian, Xuyu	Cell
11	Lgr5(+ve) Stem Cells Drive Self-Renewal in the Stomach and Build Long-Lived Gastric Units In Vitro	Disease modeling	1,115	0	354	759	Barker, Nick	Cell Stem Cell
12	SARS-CoV-2 productively infects human gut enterocytes	Basic research	1,068	0	0	1,067	Lamers, Mart M.	Science
13	In vitro expansion of single Lgr5(+) liver stem cells induced by Wnt-driven regeneration	Precision medicine	1,019	0	177	842	Huch, Meritxell	Nature
14	Patient-derived organoids model treatment response of metastatic gastrointestinal cancers	Precision medicine	1,004	0	0	1,000	Vlachogiannis, Georgios	Science
15	Organoid Cultures Derived from Patients with Advanced Prostate Cancer	Disease modeling	993	0	43	948	Gao, Dong	Cell
16	Long-Term Culture of Genome-Stable Bipotent Stem Cells from Adult Human Liver	Disease modeling	973	0	33	937	Huch, Meritxell	Cell Science
17	A Living Biobank of Breast Cancer Organoids Captures Disease Heterogeneity	Biobanking	971	0	0	967	Sachs, Norman	Cell
18	Dependency of a therapy-resistant state of cancer cells on a lipid peroxidase pathway	Basic research	968	0	0	963	Viswanathan, Vasanthi S.	Nature
19	Functional Repair of CFTR by CRISPR/Cas9 in Intestinal Stem Cell Organoids of Cystic Fibrosis Patients	Basic research	943	0	140	803	Schwank, Gerald	Cell Stem Cell
20	Kidney organoids from human iPS cells contain multiple lineages and model human nephrogenesis	Disease modeling	929	0	2	925	Takasato, Minoru	Nature

## 4 Discussion

In this study, we initially employed bibliometric analysis to examine and analyze the publications in the field of organoid research. With the tremendous potential offered by organoids, there has been a surge in research focused on their application and development. Scientific researchers and the organoids industry must comprehend and identify the trends and characteristics of these studies. This understanding aids researchers in discovering clear research directions and current challenges that need to be addressed to effectively implement interventions. Furthermore, it enables them to identify influential institutions or authors specializing in specific subfields and explore opportunities for scientific collaborations with them. Furthermore, corporations need to follow the development of science and technology (target: preclinical drug research without animal experiments) and invest in research with an eye on key clinical issues (cancer, etc.) to overcome technological barriers.

### 4.1 Characterizations of published articles

The number of publications focusing on organoids has witnessed a rapid surge over the past decade, growing nearly 70 times since 2009. The visualization of collaborative networks reveals that the majority of research in this field is conducted in developed countries like the United States, Germany, Japan, and their influential domestic institutions. China holds a dominant position among developing nations, firmly establishing its presence in organoids publications. This trend signifies the pressing need for more effective interventions in these regions. Studies of significant research value are frequently published in top international journals, with CNS (Cell, Nature, and Science) being a notable example. These journals serve as leading indicators within the life sciences domain, setting the pace for advancements in the field. The visualization of co-citation and the recognition of top-cited authors, such as Hans Clevers, Jason R. Spence, Sato Toshiro, and others, clearly demonstrate their transformative role in shaping the current concept of organoids research. Their future studies are expected to have a significant impact on advancing this field, making it crucial to closely monitor their work for the latest developments. The cooperative network clustering analysis reveals similar research focuses as those found in the visualization of co-cited journals. It is worth noting that the co-citation visualization of references provides more detailed research subcategories, offering deeper insights into the thriving research activities within the field.

### 4.2 Major developments in organoids

In 2009, Dutch scientist Hans Clevers' team successfully cultured Lgr5^+^ intestinal stem cells in vitro into three-dimensional structures with crypt-like and villous epithelial regions, opening a new chapter in the rapidly evolving study of organoids [28]. Over the past decade, a variety of organoids have been successfully constructed, and their effects in predicting the efficacy of anti-cancer drugs and guiding clinical medication have been preliminarily validated. In 2011, intestinal organoids developed from human pluripotent stem cells, and primary stem cells were successfully produced [29]. In the same year, the first retinal organoid was produced from mouse embryonic stem cells [30]. In 2013, organoids were named one of the “Top 10 Science and Technology of the Year” by the world's leading journal Science. In 2014, Memorial Sloan-Kettering Cancer Center demonstrated the first human-derived prostate tumor organoids in the laboratory [31]. With the deepening of organoid research, a variety of organoids including the small intestine [29], stomach [32], colon [26,33,34], lung [35,36], bladder [37], brain [11], liver [38], pancreas [39], kidney [40], esophagus [33], heart [41], etc. have been successfully constructed, including not only the normal organoid but also the corresponding tumor tissue organoids, and molecular function experiments and drug screening experiments have been performed based on them.

The main focus of this study is to explore the research hotspots in the field of organoids. In the analysis of institutional collaboration networks, co-citation analysis, and keyword analysis, the clustering algorithm in CiteSpace software was used to summarize and generate new clustering labels. From the results of this study, it can be seen that there are numerous research directions in the field of organoids. All analyzed clusters exceed five categories. The research hotspots mainly cover the construction of organoid models originating from various structural tissues and organs, intercellular information exchange, immune mechanisms, and cancer metastasis mechanisms. These clustering labels indicate dispersion but actually form a collaborative network. It can be clearly stated that the development of organoids involves the exploration of various complex mechanisms that are significant in practical science. In the field of basic research, animal models such as mice are highly regarded because they can simulate the metabolism and immune response of the research subject in vivo. In the field of biomedicine, animal models can replace human testing for drug toxicity. However, in reality, the results of animal model experiments cannot be directly applied to clinical medicine. Since 2009, when Dutch scientist Hans Clevers and his team successfully cultured 3D tissue structures from intestinal stem cells that could partially perform intestinal functions, the idea of using organoid models as alternatives to animal models has become feasible. This is a research area of great scientific value. In today's society, the rapid development of biotechnology and the continuous alternation of new technologies and materials have accelerated the pace of organoid research. The clustering analysis in this study also found that organoid models of various systems throughout the body have gradually been successfully constructed, and future research will be conducted on these models. However, there is still a long way to go before actual replacement of animal models can be achieved.

A study published in Science [23] in 2018 demonstrated the exceptional predictive capabilities of organoids in assessing the effectiveness of anti-cancer drugs and guiding their clinical usage. When compared to actual patient outcomes, organoids displayed remarkable performance metrics: 100% sensitivity, 93% specificity, 88% positive predictive value, and 100% negative predictive value. These findings highlight the potential of organoids in improving treatment outcomes and personalized medicine. Recently, Professor Yang's team from Guangdong Provincial People's Hospital published a study in Cell Reports Medicine [36] on predicting clinical outcomes based on organoid-based drug sensitivity testing in lung cancer. The results of the study showed that the results of organoid-based drug sensitivity testing could accurately predict the clinical efficacy of targeted or chemotherapy for lung cancer, and the overall concordance between drug sensitivity and clinical response could reach 83.33%. This study is also the largest sample size real-world study in the field of lung cancer organoids to predict the efficacy of targeted therapy and chemotherapy.

In recent years, there has been a gradual emergence of bibliometric studies related to organoids [[42], [43], [44], [45], [46], [47]]. Compared to this study, these studies have a more concentrated focus. In general, they can be divided into basic research, technological applications, and ethical concerns. The use of organoids in drug development and screening for tumor treatment has been a consistent research trend. The key question in this area is whether organoids can replace animals in testing the efficacy of drugs or for therapeutic purposes [44,48]. In terms of technological applications, Zhu et al. [47] conducted a bibliometric analysis of patents related to organoid technology, reflecting the current state of translational applications of the technology, which is undoubtedly an interesting study. In these translational research stages, the main focus has been on disease models and tumor drug development. Additionally, Ding et al. [42] conducted a bibliometric study on the ethical aspects of organoid development, which is often overlooked but extremely important. Overall, current bibliometric studies related to organoids are keeping up with the cutting-edge of research, and the aspects they focus on are also important directions currently underway or to be explored in the future. The results of this study also demonstrate this point. Although bibliometric analysis cannot provide a comprehensive analysis of existing research, it can grasp the overall direction of research, which is essential for clarifying future research focal points.

### 4.3 Uncertainty areas and future research

Organoids should be predominantly regarded as a rapidly evolving model system, encompassing mechanisms research, disease modeling, precision medicine, and the construction of biological banking. Organoid models are revolutionizing the approach to disease research in a disruptive manner. However, organoid research is resource intensive. Organoids come at a lower cost than mouse or fish models, but they are relatively more expensive when compared to traditional cell lines, fly, yeast, or worm models. Moreover, they are difficult to study at the level of whole human organs. In addition, Extracellular matrix components of organoid systems, vascularized culture protocols, and immune system establishment remain unclear. Normal and efficient communication between organs remains elusive in organoid systems. Combining organoid research with organoid on-chip technology may lead to breakthroughs, but this still relies on the compositional certainty of the extracellular matrix. Identification and resolution of immune problems, organoid maturity, and genetic stability are also guarantees for proper differentiation and transplantation of organoid cells. Future research will explore the possibility of developing co-culture systems for multiple microelements. Third, Organoid is the bridge between preclinical and clinical research. In the context of drug screening, reliable validation of the predictive value of organoid response to tumor drugs must come from ongoing clinical trials. However, attempts to do so are still lacking. Optimizing the sensitivity and robustness of drug screening platforms will be key to organoid-based personalized medicine before clinical implementation. Finally, there are still no global standardized protocols and guidance for organoid establishment and quality control programs. Collective efforts should be made to develop clear guidelines and methodologies to assess the quality and effectiveness of each organoid culture system.

### 4.4 Study limitations

Through bibliometric and visual analyses, this study provides profound insights into the global status and trends of publications on organoids, offering a unique advantage over reviews that struggle to analyze a large volume of diverse literature. However, it is important to acknowledge the limitations of our study. Firstly, there might be a bias due to the inclusion of only Web of Science databases, potentially leading to variations in the publications captured compared to other databases like PubMed, Scopus, and Embase. Secondly, the analysis process may become cumbersome as country names, plural forms of keywords, and abbreviated forms need to be combined. Lastly, the utilization of mesh subject headings in Web of Science databases that lack such headings can introduce instability in the search results.

## 5 Conclusions

Research on organoids has been developing rapidly in the last decade. Continuing to follow influential research will help to get a quick overview of trends, leading to practical and effective responses. Organoid research covers four main directions: basic research focusing on the developmental processes of stem cells and cell-cell interaction, biobanking focusing on organoid culturing, precision medicine focusing on cell therapy and drug development, and disease modeling covering pathogen analyses screening for disease-related genetic variants. Cost-saving measures, sharing of technologies, development of international standards, and conducting high-level clinical trials are the focal points for future research. Despite the remaining challenges, human organoids have great potential for clinical translational research due to their application advantages and rapid, sustained technological development.

## Funding

This study was supported by the 10.13039/100010414Health Research Board (HRB) in Ireland (Grant Reference: SS-2023-054).

## Data available statement

The data obtained from our study will be made accessible to qualified researchers by the authors.

## Institutional review board statement

Not applicable, because this article does not contain any studies with human or animal subjects.

## Informed consent statement

Not applicable.

## CRediT authorship contribution statement

**Huanyu Li:** Writing – original draft, Methodology, Formal analysis, Data curation. **Daofeng Wang:** Writing – review & editing, Visualization, Methodology, Data curation. **Cheong Wong Ho:** Writing – review & editing, Methodology. **Dan Shan:** Writing – review & editing, Supervision, Funding acquisition, Conceptualization.

## Declaration of competing interest

The authors declare that they have no known competing financial interests or personal relationships that could have appeared to influence the work reported in this paper.

## Figures and Tables

**figs1:**
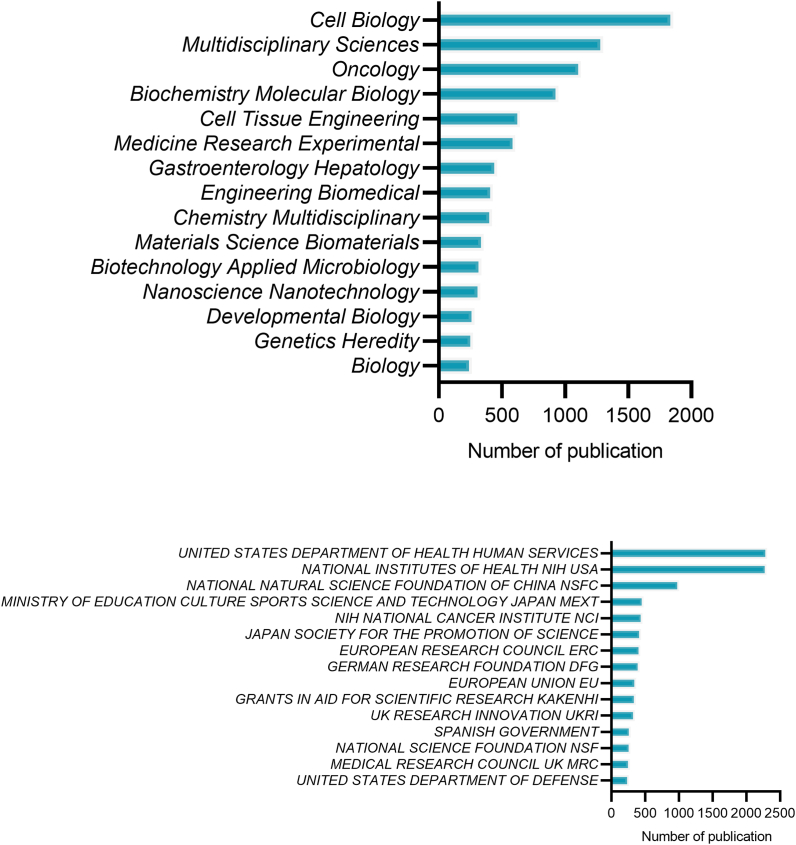


## References

[1] Wang Y., Meng L., Meng S., Huang L., Luo S., Wu X., Gong X. (2023). Flotillin-1 enhances radioresistance through reducing radiation-induced DNA damage and promoting immune escape via STING signaling pathway in non-small cell lung cancer. Cancer Biol. Ther..

[2] Gu J.J., Li H.X., Wei W., Sun X.L., Li B.C., Chen Y., Li J., Gu X. (2023). Bone marrow mesenchymal stem cell transplantation alleviates radiation-induced myocardial fibrosis through inhibition of the TGF-β1/Smad2/3 signaling pathway in rabbit model. Regen. Ther..

[3] Haloi P., Lokesh B.S., Chawla S., Konkimalla V.B. (2023). Formulation of a dual drug-loaded nanoparticulate co-delivery hydrogel system and its validation in rheumatoid arthritis animal model. Drug Deliv..

[4] Zheng R., Lin C., Mao Y., Jin F. (2023). miR-761-hepcidin/Gpx4 pathway contribute to unexplained liver dysfunction in polycystic ovary syndrome by regulating liver iron overload and ferroptosis. Gynecol. Endocrinol..

[5] Zheng Y., Wu J., Chen H., Lin D., Chen H., Zheng J., Xia H., Huang L., Zeng C. (2023). KLF4 targets RAB26 and decreases 5-FU resistance through inhibiting autophagy in colon cancer. Cancer Biol. Ther..

[6] Huang Q., An R., Wang H., Yang Y., Tang C., Wang J., Yu W., Zhou Y., Zhang Y., Wu D. (2023). Aggravated pneumonia and diabetes in SARS-CoV-2 infected diabetic mice. Emerg. Microb. Infect..

[7] Kamb A. (2005). What's wrong with our cancer models? Nature reviews. Drug Discov..

[8] Caponigro G., Sellers W.R. (2011). Advances in the preclinical testing of cancer therapeutic hypotheses. Nat. Rev. Drug Discov..

[9] Cheon D.J., Orsulic S. (2011). Mouse models of cancer. Annu. Rev. Pathol..

[10] Kim J., Koo B.K., Knoblich J.A. (2020). Human organoids: model systems for human biology and medicine. Nat. Rev. Mol. Cell Biol..

[11] Lancaster M.A., Renner M., Martin C.A., Wenzel D., Bicknell L.S., Hurles M.E., Homfray T., Penninger J.M., Jackson A.P., Knoblich J.A. (2013). Cerebral organoids model human brain development and microcephaly. Nature.

[12] Jo J., Xiao Y., Sun A.X., Cukuroglu E., Tran H.D., Göke J., Tan Z.Y., Saw T.Y., Tan C.P., Lokman H. (2016). Midbrain-like organoids from human pluripotent stem cells contain functional dopaminergic and neuromelanin-producing neurons. Cell Stem Cell.

[13] Yan H.H.N., Siu H.C., Law S., Ho S.L., Yue S.S.K., Tsui W.Y., Chan D., Chan A.S., Ma S., Lam K.O. (2018). A comprehensive human gastric cancer organoid biobank captures tumor subtype heterogeneity and enables therapeutic screening. Cell Stem Cell.

[14] Bartfeld S., Bayram T., van de Wetering M., Huch M., Begthel H., Kujala P., Vries R., Peters P.J., Clevers H. (2015). In vitro expansion of human gastric epithelial stem cells and their responses to bacterial infection. Gastroenterology.

[15] Tang F., Dai W.B., Li X.L., Turghun D., Huang H., Fan Y.Q. (2021). Publication trends and hot spots in femoroacetabular impingement research: a 20-year bibliometric analysis. J. Arthroplasty.

[16] Chen C., Song M. (2019). Visualizing a field of research: a methodology of systematic scientometric reviews. PLoS One.

[17] Shen W., Shao A., Zhou W., Lou L., Grzybowski A., Jin K., Ye J. (2023). Retinogenesis in a dish: bibliometric analysis and visualization of retinal organoids from 2011 to 2022. Cell Transplant..

[18] Wang Z., He X., Qiao H., Chen P. (2020). Global trends of organoid and organ-on-a-chip in the past decade: a bibliometric and comparative study. Tissue Eng. Part A.

[19] Bastian S., Ippolito J.A., Lopez S.A., Eloy J.A., Beebe K.S. (2017). The use of the h-index in academic orthopaedic surgery. J. Bone Joint Surg. Am..

[20] Durieux V., Gevenois P.A. (2010). Bibliometric indicators: quality measurements of scientific publication. Radiology.

[21] Wang K., Xing D., Dong S., Lin J. (2019). The global state of research in nonsurgical treatment of knee osteoarthritis: a bibliometric and visualized study. BMC Muscoskel. Disord..

[22] Clevers H. (2016). Modeling development and disease with organoids. Cell.

[23] Vlachogiannis G., Hedayat S., Vatsiou A., Jamin Y., Fernández-Mateos J., Khan K., Lampis A., Eason K., Huntingford I., Burke R. (2018). Patient-derived organoids model treatment response of metastatic gastrointestinal cancers. Science.

[24] Qian X., Nguyen H.N., Song M.M., Hadiono C., Ogden S.C., Hammack C., Yao B., Hamersky G.R., Jacob F., Zhong C. (2016). Brain-region-specific organoids using mini-bioreactors for modeling ZIKV exposure. Cell.

[25] Sachs N., de Ligt J., Kopper O., Gogola E., Bounova G., Weeber F., Balgobind A.V., Wind K., Gracanin A., Begthel H. (2018). A living biobank of breast cancer organoids captures disease heterogeneity. Cell.

[26] van de Wetering M., Francies H.E., Francis J.M., Bounova G., Iorio F., Pronk A., van Houdt W., van Gorp J., Taylor-Weiner A., Kester L. (2015). Prospective derivation of a living organoid biobank of colorectal cancer patients. Cell.

[27] Eyre-Walker A., Stoletzki N. (2013). The assessment of science: the relative merits of post-publication review, the impact factor, and the number of citations. PLoS Biol..

[28] Sato T., Vries R.G., Snippert H.J., van de Wetering M., Barker N., Stange D.E., van Es J.H., Abo A., Kujala P., Peters P.J., Clevers H. (2009). Single Lgr5 stem cells build crypt-villus structures in vitro without a mesenchymal niche. Nature.

[29] McCracken K.W., Howell J.C., Wells J.M., Spence J.R. (2011). Generating human intestinal tissue from pluripotent stem cells in vitro. Nat. Protoc..

[30] Silva G.A., Silva N.F., Fortunato T.M. (2011). Stem cell and tissue engineering therapies for ocular regeneration. Curr. Stem Cell Res. Ther..

[31] Gao D., Vela I., Sboner A., Iaquinta P.J., Karthaus W.R., Gopalan A., Dowling C., Wanjala J.N., Undvall E.A., Arora V.K. (2014). Organoid cultures derived from patients with advanced prostate cancer. Cell.

[32] Byrne A.T., Alférez D.G., Amant F., Annibali D., Arribas J., Biankin A.V., Bruna A., Budinská E., Caldas C., Chang D.K. (2017). Interrogating open issues in cancer medicine with patient-derived xenografts. Nat. Rev. Cancer.

[33] Sato T., Stange D.E., Ferrante M., Vries R.G., Van Es J.H., Van den Brink S., Van Houdt W.J., Pronk A., Van Gorp J., Siersema P.D., Clevers H. (2011). Long-term expansion of epithelial organoids from human colon, adenoma, adenocarcinoma, and Barrett's epithelium. Gastroenterology.

[34] Fujii M., Shimokawa M., Date S., Takano A., Matano M., Nanki K., Ohta Y., Toshimitsu K., Nakazato Y., Kawasaki K. (2016). A colorectal tumor organoid library demonstrates progressive loss of niche factor requirements during tumorigenesis. Cell Stem Cell.

[35] Dye B.R., Hill D.R., Ferguson M.A., Tsai Y.H., Nagy M.S., Dyal R., Wells J.M., Mayhew C.N., Nattiv R., Klein O.D. (2015). In vitro generation of human pluripotent stem cell derived lung organoids. Elife.

[36] Wang H.M., Zhang C.Y., Peng K.C., Chen Z.X., Su J.W., Li Y.F., Li W.F., Gao Q.Y., Zhang S.L., Chen Y.Q. (2023). Using patient-derived organoids to predict locally advanced or metastatic lung cancer tumor response: a real-world study. Cell Rep. Med..

[37] Xiao K., Peng S., Lu J., Zhou T., Hong X., Chen S., Liu G., Li H., Huang J., Chen X., Lin T. (2023). UBE2S interacting with TRIM21 mediates the K11-linked ubiquitination of LPP to promote the lymphatic metastasis of bladder cancer. Cell Death Dis..

[38] Broutier L., Mastrogiovanni G., Verstegen M.M., Francies H.E., Gavarró L.M., Bradshaw C.R., Allen G.E., Arnes-Benito R., Sidorova O., Gaspersz M.P. (2017). Human primary liver cancer-derived organoid cultures for disease modeling and drug screening. Nat. Med..

[39] Boj S.F., Hwang C.I., Baker L.A., Chio I.I.C., Engle D.D., Corbo V., Jager M., Ponz-Sarvise M., Tiriac H., Spector M.S. (2015). Organoid models of human and mouse ductal pancreatic cancer. Cell.

[40] Freedman B.S., Brooks C.R., Lam A.Q., Fu H., Morizane R., Agrawal V., Saad A.F., Li M.K., Hughes M.R., Werff R.V. (2015). Modelling kidney disease with CRISPR-mutant kidney organoids derived from human pluripotent epiblast spheroids. Nat. Commun..

[41] Zhang Y.S., Arneri A., Bersini S., Shin S.R., Zhu K., Goli-Malekabadi Z., Aleman J., Colosi C., Busignani F., Dell'Erba V. (2016). Bioprinting 3D microfibrous scaffolds for engineering endothelialized myocardium and heart-on-a-chip. Biomaterials.

[42] Ding L., Xiao Z., Gong X., Peng Y. (2022). Knowledge graphs of ethical concerns of cerebral organoids. Cell Prolif..

[43] Ding Z., Tang N., Huang J., Cao X., Wu S. (2023). Global hotspots and emerging trends in 3D bioprinting research. Front. Bioeng. Biotechnol..

[44] Li M., Yuan Y., Zou T., Hou Z., Jin L., Wang B. (2023). Development trends of human organoid-based COVID-19 research based on bibliometric analysis. Cell Prolif..

[45] Tan S., Deng J., Deng H., Lu L., Qin Z., Liu Y., Tang L., Li Z. (2023). Global cluster analysis and network visualization in organoids in cancer research: a scientometric mapping from 1991 to 2021. Front. Oncol..

[46] Zhang M.M., Yang K.L., Cui Y.C., Zhou Y.S., Zhang H.R., Wang Q., Ye Y.J., Wang S., Jiang K.W. (2021). Current trends and research topics regarding intestinal organoids: an overview based on bibliometrics. Front. Cell Dev. Biol..

[47] Zhu L., Fan Y., Huang X., Chen T., Xu X., Xu F., Gong Y., Chen P. (2022). Patent bibliometric analysis for global trend of organoid technologies in the past decade. iScience.

[48] Shuoxin Y., Shuping W., Xinyue Z., Tao Z., Yuanneng C. (2023). Progress of research on tumor organoids: a bibliometric analysis of relevant publications from 2011 to 2021. Front. Oncol..

